# Serum Levels of Trace Elements (Magnesium, Iron, Zinc, Selenium, and Strontium) are Differentially Associated with Surrogate Markers of Cardiovascular Disease Risk in Patients with Rheumatoid Arthritis

**DOI:** 10.1007/s12011-024-04434-8

**Published:** 2024-10-30

**Authors:** Enric Vera, Joan-Carles Vallvé, Victòria Linares, Silvia Paredes, Daiana Ibarretxe, Montserrat Bellés

**Affiliations:** 1 Laboratory of Toxicology and Environmental Health, School of Medicine, Universitat Rovira I Virgili, Reus, Spain; 2 Unitat de Recerca en Lípids I Arteriosclerosi, Universitat Rovira I Virgili, Sant Llorenç 21, 43201 Reus, Spain; 3 Institut d’Investigació Sanitària Pere Virgili (IISPV), Reus, Spain; 4 Unitat de Medicina Vascular I Metabolisme. Hospital Universitari Sant Joan de Reus, Reus, Spain; 5 Centro de Investigación Biomédica en Red de Diabetes y Enfermedades Metabólicas Asociadas (CIBERDEM), Madrid, Spain; 6 Secció de Reumatologia. Hospital Universitari Sant Joan de Reus, Reus, Spain

**Keywords:** Rheumatoid Arthritis, Cardiovascular Disease, Atherosclerosis, Trace Elements

## Abstract

Rheumatoid arthritis (RA) is associated with an increased risk of cardiovascular disease (CVD), which is attributed to inflammatory processes that accelerate atherosclerosis. Therefore, the exploration of novel biomarkers association is needed. This study investigated the associations between serum trace elements (Fe, Zn, Mg, Se, and Sr) and surrogate markers of CVD in 219 RA patients and compared them with those with metabolic disorders (MetD, *n* = 82) and control participants (*n* = 64). Surrogate markers included carotid intima-media thickness (cIMT), carotid plaque presence (cPP), pulse wave velocity (PWV), distensibility (DIST), and the augmentation index (AIx). RA patients displayed heightened inflammatory markers, increased arterial stiffness and thickness, and elevated CV risk factors. Compared with those in control participants, Se levels in RA patients were lower, regardless of sex. Women and men with RA had lower Sr and Mg levels than those with MetD, respectively. Backward regression models demonstrated inverse associations of Sr and Zn with cIMT in men with RA and those with MetD, respectively. In RA patients, Sr and Zn were predictors of an increased AIx, with sex-specific associations. Increased Fe levels were associated with an increased AIx in women with MetD. Fe and Zn were predictors of increased cIMT in control participants, with sex-specific associations. Serum trace elements are independently associated with surrogate markers of CVD in patients with RA, highlighting their potential role in CV risk assessment. Prospective studies are essential for validating these associations and establishing optimal trace element levels for managing CVD risk in patients with RA.

**Supplementary Information:**

The online version contains supplementary material available at 10.1007/s12011-024-04434-8.

## Introduction

Rheumatoid arthritis (RA) is a systemic chronic autoimmune disease characterized by symmetrical inflammatory polyarthritis that mainly affects peripheral joints, causing irreversible and progressive joint damage [1]. The resulting deformities gradually lead to physical disability and impaired quality of life. With a prevalence ranging between 0.5 and 1%, RA is one of the most common chronic inflammatory diseases in the general population [2–6].

Patients with RA experience an increased risk of cardiovascular disease (CVD), estimated to be 50% greater than that of the general population, with a mortality rate 1.5 times greater [7]. The increased risk is partly explained by the chronic inflammatory state of RA, where the inflammatory process plays a pivotal role in atherosclerosis development. Traditional CV risk factors alone neither fully explain the accelerated atherosclerosis process nor the increase in CV risk observed in these patients, given the complexity of the mechanisms underlying the progression of atherosclerosis in RA patients [8]. Therefore, new biomarkers that elucidate novel mechanisms are needed to accurately assess this risk. To better understand the mechanisms involved in RA, these biomarkers must be specific and sensitive to RA, as other metabolic disorders, such as diabetes, dyslipidemia, and hypertension—common in RA patients—are significant contributors to cardiovascular risk. These patients with metabolic disorders exhibit a lower degree of inflammation, originating from different causes compared to the high-grade autoimmune inflammation seen in RA. Differentiating between these aspects could provide deeper insights into the cardiovascular risk associated with RA.

Atherosclerosis is a common condition that can be measured at a subclinical level before becoming clinically evident. Detecting subclinical atherosclerosis is crucial, as it enables early interventions to prevent the progression of the disease and reduce the risk of cardiovascular events. Various noninvasive techniques can be used to evaluate the presence and extent of subclinical atherosclerosis, including carotid intima-media thickness (cIMT), carotid plaque presence (cPP) and several arterial stiffness measures, such as pulse wave velocity (PWV), distensibility (DIST) and the augmentation index (AIx). All these variables are good predictors of CVD and are considered surrogate markers of CVD [9–12].

Addressing modifiable clinical risk factors is crucial for mitigating the burden of chronic diseases. In this context, trace elements, including magnesium (Mg), iron (Fe), zinc (Zn), selenium (Se), and strontium (Sr), play essential roles in maintaining optimal body functions. Thus, they contribute significantly to many cellular processes, serve as essential cofactors for numerous metabolic enzymes and are indispensable for maintaining energy homeostasis [13]. However, their imbalance or deficiency has been implicated in several chronic diseases [14–22].

Several studies on trace element levels among RA patients have yielded inconclusive results. For instance, deficiencies in Mg, Fe, Zn and Se have been reported in patients with RA, suggesting potential roles for these trace elements in this disease [23–27]. However, increased levels of Mg, Fe and Sr have also been described in RA patients [28, 29]. These trace elements are also implicated in several processes related to the pathophysiology of atherosclerosis. For instance, Mg modulates the production of proinflammatory cytokines [15, 30, 31], and Zn and Se exhibit antioxidant, anti-inflammatory and immunomodulatory functions [24, 32–34]. Furthermore, Wang et al. reported an inverse correlation between inflammatory markers and serum Fe levels in RA patients [35]. However, the specific associations between serum levels of Mg, Fe, Zn, Se and Sr and surrogate markers of CVD, especially in RA patients, are less studied and remain unclear. This gap in knowledge warrants further investigation into the potential role of trace elements in the increased cardiovascular risk observed in RA patients.

To contribute to the diagnosis and prognosis of CVD in RA patients, the present study aimed to evaluate, in a cohort of 219 patients with RA, the serum concentrations of five crucial trace elements (Mg, Fe, Zn, Se and Sr) and their potential associations with CV risk using diverse surrogate markers of CVD. By comparing these findings with those from patients with metabolic disturbances (*n* = 82) who have a high cardiovascular risk, as well as with a control group (*n* = 64), the study aimed to discern the specificity of these potential associations. Additionally, we aimed to assess the potential clinical implications of these findings for the effective management of RA.

## Methods and Patients

### Patients

The RA cohort in this cross-sectional study has been previously characterized and comprises individuals who visited the University Hospital Sant Joan de Reus for outpatient appointments and met the American College of Rheumatology’s 1987 classification criteria for RA [36]. Individuals over the age of 80 or under the age of 18, those with concurrent acute illnesses, and those with a change in their disease diagnosis were excluded from the study. A total of 219 patients between the ages of 18 and 80 were enrolled in the study, and blood samples were collected on the same day as their medical appointments. Additionally, 82 patients who voluntarily participated in the study and were being treated at the *Vascular Medicine and Metabolism Unit* of our hospital for lipid metabolism disorders and related conditions, such as obesity, type 2 diabetes mellitus, and metabolic syndrome, were included and composed the *metabolic disorder* (MetD) *group*. Furthermore, 64 control subjects free of medical conditions were recruited from the hospital’s personnel pool. The study was approved by the Clinical Research Ethics Committee of Hospital Sant Joan de Reus (patients with RA: 11–04-28/4proj5, control participants and patients with metabolic disorders: CEIm: 222/2020), and all participants provided written informed consent. The study was conducted in accordance with the guidelines of our institution and the Declaration of Helsinki.

### Clinical Evaluation and Laboratory Measurements

Information on the presence of classical CV risk factors (smoking, hypertension, diabetes, and dyslipidemia), as well as the history of CV events and drug consumption, was collected. Clinical measures such as body weight, height, body mass index (BMI), waist circumference (WC), systolic blood pressure (SBP) and diastolic blood pressure (DBP) were assessed. In addition, we performed physical examinations on the joints of patients, including the numbers of swollen and tender joints (SJC and TJC, respectively) [12].

Blood samples were collected from all participants following a minimum fasting period of 12 h. Upon collection, samples were immediately processed at our research institute’s Biobank. Whole blood was subjected to centrifugation at 3000 rpm for 10 min to separate serum or plasma, depending on the specific analytical determination required. The resulting serum and plasma samples were then aliquoted and stored at − 80 °C in our Biobank for subsequent analysis. Enzymatic and standard methods were employed for the analytical assessments. Lipid profile (total cholesterol, LDL cholesterol, HDL cholesterol, and triglycerides) and glucose were measured in plasma while rheumatoid factor (RF), and anti-citrullinated cyclic peptides (anti-CCP) were measured in serum. Furthermore, the inflammatory markers such erythrocyte sedimentation rate (ESR), C-reactive protein (CRP), and fibrinogen were measured in blood, serum, and plasma respectively. RF positivity (RF +) was defined as RF values > 20, and anti-CCP positivity (anti-CCP +) was defined as anti-CCP values > 3.

### Trace Element Measurements

One hundred microliters of each serum sample were digested in 100 µL of HNO3 (65% Suprapur, Merck, Darmstadt, Germany) in a Milestone Start D microwave digestion system (Sorisole, Italy). They were diluted in 5 mL of ultrapure water and kept at − 20 °C until analysis. The concentrations of Mg, Fe, Zn, Se and Sr in the serum were determined by inductively coupled plasma‒mass spectrometry (ICP-MS) (Perkin-Elmer Elan 6000, Woodbridge, ON, Canada) following the manufacturer’s instructions. The accuracy of the analytical procedures was checked by performing the analyses in duplicate. Quality control was assured by the analysis of a certified reference material, Seronorm™ Trace elements whole blood L-2 (SERO, Billingstad, Norway). Trace elements were not found in devices used for sample collection, storage, preparation, or analysis. The limits of detection (LODs) were 0.1 µg/mL for Mg, 0.25 µg/mL for Fe, 0.2 µg/mL for Zn, 0.05 µg/mL for Se, and 0.005 µg/mL for Sr.

### Ultrasound Evaluation of Carotid Intima–Media Thickness and Arterial Stiffness

cIMT measures the thickness of the innermost two layers of the carotid artery wall, with higher values of cIMT indicating an increased presence of atherosclerosis. To measure cIMT, we used a My Lab 60 X-Vision sonographer (Esaote SpA, Genova, Italy) with a linear array ultrasound probe small parts broadband transducer (5–12 MHz). We identified and digitally recorded the far wall of the common carotid artery (1 cm proximal to the bifurcation), the bifurcation, and the internal carotid artery (1 cm distal to the bifurcation) of the left and right carotid arteries. In vivo measurements of cIMT were performed at common carotid arteries using QIMT© radiofrequency (RF) image processing software (Esaote SpA, Genova, Italy). To reduce observer variability, a single operator obtained and measured the images. We averaged the measurements of the left and right common carotid arteries to obtain the mean cIMT. Following the Mannheim consensus, we defined carotid plaque presence (cPP) as a focal structure encroaching into the arterial lumen by a minimum of 0.5 mm, or 50% of the surrounding IMT value, or displaying a thickness greater than 1.5 mm measured from the intima-lumen interface to the media-adventitia interface [37]. The presence and number of carotid plaques are clinically relevant as it serves as a significant predictor for CV events, thereby helping in the risk stratification for CVD.

Arterial stiffness can be accurately assessed by measurements of the PWV, DIST, and the AIx. PWV, DIST, and the AIx were measured directly at both common carotid arteries using an ultrasound linear probe (5–12 MHz) as a tonometer and analyzed in vivo with Quality Arterial Stiffness (QAS©) radiofrequency software (Esaote SpA, Genova, Italy). RF signal-based vascular ultrasound from Esaote employs RF signal-based technology and includes QAS measurements. The RF signal is a reflected ultrasound signal that is captured by the transducer and converted into an electric signal preserving all the characteristics of the acoustic wave in terms of amplitude and phase. Local arterial stiffness is estimated as systo-diastolic changes in arterial diameter/area over systo-diastolic changes in distending pressure (pulse pressure) [11]. Maximum and minimum carotid diameters were acquired using the attained distension curves, and vascular stiffness parameters were calculated after calibration for blood pressure [38, 39]. The final values were the median measurements of the right and left common carotid arteries [40, 41]. The examination was performed according to standardized measurements [42]. PWV measures the speed at which the arterial pressure wave travels through the arteries and is obtained from brachial blood pressure and accurate measurements of the diameter and change in diameter of carotid arteries. Carotid DIST assesses the ability of the artery to expand and contract in response to intravascular volume expansion caused by left ventricular systole, reflecting carotid wall elasticity. Higher PWV and DIST values indicate greater and lesser arterial stiffness, respectively. The AIx was measured by pulse wave analyses and local pressure. The AIx measures the reflection of the pressure wave from the peripheral arteries back to the central arteries during the cardiac cycle, providing insight into arterial stiffness and peripheral resistance. Higher AIx values indicate increased arterial stiffness and a greater burden on the heart during systolic ejection [11].

### Statistical Methods

Continuous normally distributed variables are expressed as the mean and standard deviation (SD), while continuous nonnormally distributed variables are expressed as the median and interquartile range (IQR). Categorical variables are expressed as the percentage and number of individuals. To assess differences between normally distributed, nonnormally distributed, and categorical variables, *t* tests, Kruskal‒Wallis tests, and chi‒square tests were used.

Multivariate linear regression models were adjusted to investigate the independent associations between trace element levels and surrogate markers of CVD (cIMT, PWV, DIST, and the AIx). Initially, all models were adjusted by age, sex, BMI, SBP, and DBP. To identify the most influential predictors, we utilized a backward elimination approach, systematically removing less significant variables from the model. This statistical method enhances the robustness of our analysis, ensuring that the final model includes only the most pertinent predictors for a more accurate evaluation of their impact on cardiovascular health indicators [43]. Applying this approach, the variables included in the linear regression models were as follow: for cIMT, the method selected, age, BMI, SBP, and DBP for Sr, Zn, Mg, and Se, while age, BMI, and SBP were included for Fe. For PWV, age, sex, BMI, SBP, and DBP were selected for Mg, Fe, Zn, and Se, whereas only age was selected for Sr. In the case of AIx, age, BMI, SBP, and DBP were included for all elements. Finally, for DIST, the method selected age, sex, BMI, SBP, and DBP for all elements (see figure legends).

Trace element variables were analysed without transformation when no issues with normality were identified. However, when the regression analysis revealed nonnormality of residuals (assessed by QQ plots) or heterogeneity of variance (as visualized by graphs of standardized residuals vs. predicted values), the trace element variables were log-transformed. This transformation was applied to all models for Se and Sr, the models for Fe when evaluating the association with AIx in the group of women with MetD and the models for Zn when evaluating the association with cIMT in the group of men with MetD (see figure legends). The association between each trace element variable and cPP was evaluated with multivariate logistic regression analysis. Models of logistic regressions were calculated using serum concentration values of Mg, Fe and Zn, and using logarithmic values of Se and Sr and adjusted for age, sex, BMI, SBP and DBP. Linear and logistic regression analyses were carried out in the overall cohort and stratified by sex. Differences were considered significant at the *P* < 0.05 level. Statistical analysis was performed using the Statistical Package for the Social Sciences version 27.0 (SPSS Inc., IBM Corp, Chicago, IL, USA) program.

## Results

### Study Group Characteristics

The general characteristics of our study population groups are presented in Table 1. Briefly, the distribution of sex did not differ significantly among the three groups. However, it is worth noting that patients with RA and MetD were significantly older and had higher BMI, WC, SBP, DBP, and triglyceride (TG) than those in the control group. There were no significant differences in terms of high-density lipoprotein cholesterol (HDLc) levels, but RA patients had lower levels of low-density lipoprotein cholesterol (LDLc) compared to MetD patients. In terms of inflammatory markers, RA patients exhibited elevated ESR, CRP, and fibrinogen levels compared to those in both the control group and MetD patients. Additionally, within the group of RA patients, 23.3% achieved remission, 19.2% showed low disease activity, 46.1% had moderate disease activity, and 11.4% had high disease activity. Most RA patients (74%) were treated with conventional disease-modifying antirheumatic drugs (DMARDs), 58.4% with nonsteroidal anti-inflammatory drugs (NSAIDs), and 52.1% with corticoids (CSs), while 21.0% received biological treatment.

**Table 1:** General characteristics of the cohort

	Control (*n* = 64)	MetD (*n* = 82)	RA (*n* = 219)	*P*-value
Characteristics of the groups				
Sex – female (%, *n*)	78.1%, 50	70.7%, 58	65.3%, 165	0.136
Age (years, S.D.)	51.2 (9.1)^a,b,c^	62.8 (11.9) ^a,b,c^	57.6 (12.1) ^a,b,c^	< 0.001
BMI (kg/m^2^, IQR)	24.6 (21.6–27.5)^a,b^	26.6 (23.3–29.8)^a^	26.9 (23.5–30.9)^b^	< 0.001
Waist circumference (cm, S.D.)	86.3 (10.3)^a,b^	95.5 (13.9)^a^	92.2 (14.8)^b^	< 0.001
SBP (mmHg, IQR)	120 (110–130)^a,b^	135 (122.5–150)^a^	135 (120–150)^b^	< 0.001
DBP (mmHg, IQR)	72.5 (65–80)^a,b^	80 (75–88.2)^a^	80 (89–72)^b^	< 0.001
LDL cholesterol (mg/dL, IQR)	115 (100.5–130.7)	123 (106.7–140.7)^c^	114 (99–135)^c^	0.082
HDL cholesterol (mg/dL, IQR)	69 (59.2–77.7)	62 (48.5–75.2)	66 (53–76)	0.147
Triglycerides (mg/dL, IQR)	76 (57–95.7)^a,b^	93.5 (75–142.2)^a^	93 (69–128)^b^	< 0.001
Glucose (mg/dL, IQR)	91.50 (85.2–97.7)^a^	94.5 (89.7–102)^a,c^	89 (82–99)^c^	0.001
Current smoker (%,n)	15.6%, 10	9.8%, 8^c^	26.9%, 59^c^	0.003
Hypertension (%,n)	0%, 0	56.1%, 46	45.7%, 100	0.107
Diabetes mellitus (%,n)	0%, 0	6.1%, 5	11.4%, 25	0.170
Dyslipidaemia (%,n)	0%, 0	63.4%, 52	40.6%, 89	< 0.001
Disease features				
Disease duration (years, IQR)	-	-	7 (2–13)	-
DAS28-ESR (median, IQR)	-	-	3.48 (2.66–4.38)	-
Disease activity (%,n)				
Remission (< 2,6)	-	-	23.3%, 51	-
Low (2,6 to ≤ 3,2)	-	-	19.2%, 42	-
Moderate (3,2 to ≤ 5,1)	-	-	46.1%, 101	-
High (> 5,1)	-	-	11.4%, 25	-
HAQ (median, IQR)	-	-	0.38 (0–0.88)	-
RF + (%, n)			75.8%, 166	-
ACPA + (%, n)	-	-	74.7%, 162	-
ESR (mm/h, IQR)	9.50 (5–14.7)^a,b,c^	16 (8.75–27.2)^a,b,c^	31 (19–50)^a,b,c^	< 0.001
CRP (mg/dL, IQR)	0.12 (0.06–0.20)^b^	0.12 (0.07–0.25)^c^	0.50 (0.20–1)^b,c^	< 0.001
Fibrinogen (mg/dL, S.D.)	313.3 (60.8)^b^	344.8 (71.9)^c^	444.8 (95.1)^b,c^	< 0.001
Treatments (%, *n*)				
RA treatment				
csDMARDs	-	-	74%, 162	-
Biological agent	-	-	21%, 46	-
NSAIDs	-	-	58.4%, 128	-
CS (mean dose: 2,91 mg)	-	-	52.1%, 115	-
Hypertension treatment	0%, 0	39%, 32	35.2%, 77	0.487
Dyslipidaemia treatment	0%, 0	28% (28.4%), 23	17.8%, 39	0.050
Diabetes Mellitus treatment	0%, 0	0%, 0	8.2%, 18	-
US measurements and CV events				
cIMT (μm)	578.2 (515–632.2)^a,b^	608.5 (556.7–730)^a^	630 (570–705.5)^b^	< 0.001
PWV (m/s)	6.90 (5.88–8.11)^a,b^	8.30 (7.01–9.71)^a^	7.96 (6.88–9.64)^b^	< 0.001
Plaque presence (%, *n*)	0%, 0	34.1%, 28	42.9%, 94	0.246
Previous CV events (%, *n*)	0%, 0	0%, 0	9.6%, 21	-

General characteristics of the cohort: Description of the general characteristics, disease features and treatments of the control participants (C), metabolic disease (MetD) patients, and rheumatoid arthritis (RA) patients. *P-*values < 0.05 were considered to indicate statistical significance. ^a^statistical comparison between the control group and metabolic group; ^b^statistical comparison between the control group and RA group; ^c^ statistical comparison between the metabolic group and RA group

*n* number of individuals, *BMI* body mass index, *IQR* interquartile range, *SBP* systolic blood pressure, *DBP* diastolic blood pressure, *LDL* low-density lipoprotein, *HDL* high-density lipoprotein, *HAQ* health assessment questionnaire, *RF* + rheumatoid factor positive, *ACPA* anti-citrullinated protein antibody, *ESR* erythrocyte sedimentation rate, *CRP* c-reactive protein, *csDMARD* conventional synthetic disease-modifying antirheumatic drug, *NSAID* non-steroidal anti-inflammatory drugs, *CS* corticoids, *CV* cardiovascular, *cIMT* carotid intima-media thickness, *PWV* pulse wave velocity

Furthermore, ultrasonography analyses revealed that both RA and MetD patients had significantly greater cIMT and PWV compared to the control group, with no significant differences between the two patient groups. Additionally, RA patients exhibited significantly greater AIx values than patients in both the MetD and control groups. However, there were no differences observed in terms of arterial DIST among the three groups. Interestingly, there was no significant difference in the prevalence of cPP between the two groups of patients.

### Serum Trace Element Concentrations

**Fig. 1:**
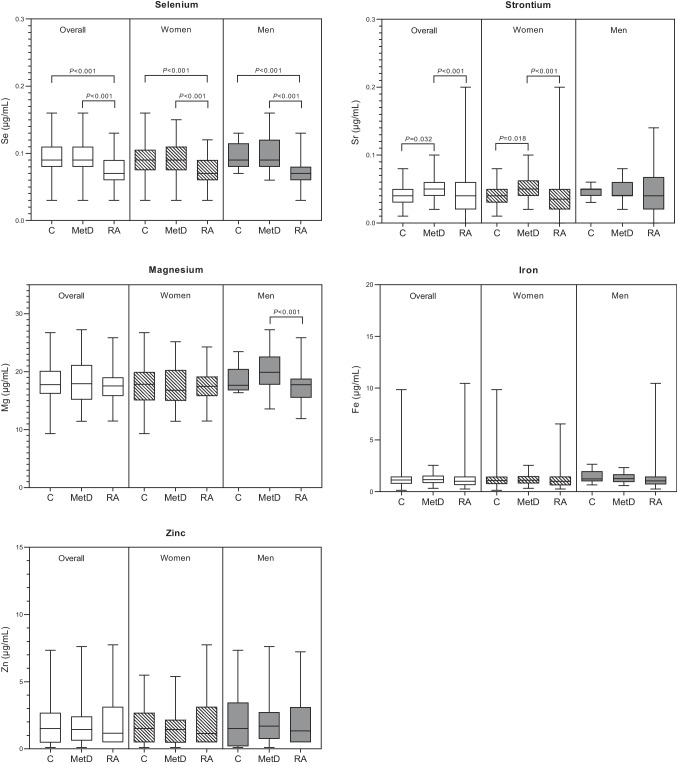
Serum trace element concentrations in the overall population and stratified by sex in control participants (C), metabolic disease (MetD) patients, and rheumatoid arthritis (RA) patients. Box plots for the nonparametric tests evaluating the differences in trace element concentrations between groups. *P*-values < 0.05 were considered to indicate statistical significance

The serum concentrations of trace elements among the groups are depicted in Fig. 1. Our findings indicate that RA patients exhibit significantly lower serum concentrations of Se than patients in both the control and MetD groups. When stratified by sex, this pattern of lower Se levels in RA patients remained consistent for both females and males in comparison to their respective control and MetD groups. Furthermore, RA patients had significantly lower levels of Sr than MetD patients, although no significant differences were observed when compared to the control group. However, when stratified by sex, it is noteworthy that only women with RA exhibited significantly lower levels of Sr than did MetD patients. Additionally, MetD patients had significantly greater levels of Sr than control participants, and this difference was observed only in women. In terms of serum Mg concentrations, no significant differences were observed among the three groups when considering the entire population. Nevertheless, upon stratification by sex, our analysis revealed that men with RA had significantly lower levels of Mg compared to those with MetD. No differences were found among women in any of the aforementioned comparisons. Moreover, we found no significant differences in the serum Fe and Zn concentrations between the RA group and the control or MetD groups, regardless of sex.

Furthermore, our Spearman correlation analyses showed that, in RA patients, Sr was correlated with age (r = 0.25, *P* = 0.0001), WC (r = 0.16,* P* = 0.016), and DBP (r = 0.16, *P* = 0.021) and Fe was correlated with DBP (r = 0.15, *P* = 0.024). We also observed a correlation between Zn and age (r =  − 0.24, *P* = 0.028) in the MetD group and between Mg and Se and age (r =  − 0.33, *P* = 0.009; r =  − 0.37, *P* = 0.003, respectively), and between Zn and SBP (r = 0.30, *P* = 0.021) and DBP (r = 0.37, *P* = 0.003) in the control group. Moreover, Fe levels in MetD patients were significantly greater in smokers (*P* = 0.022) and ex-smokers (*P* = 0.090) than in nonsmokers, and Zn concentrations in the control group were lower in ex-smokers than in nonsmokers and smokers (*P* = 0.012 and *P* = 0.01, respectively). No differences were observed in RA patients regarding smoking habits (Online Resource 1). Additionally, we observed that RA and MetD patients with cPP had higher levels of Sr (*P* = 0.006) and lower levels of Mg (*P* = 0.049), respectively (Online Resource 2).

### Associations of Serum Trace Elements with Surrogate Markers of CVD

Spearman correlation analyses of the ultrasonographic variables revealed that Sr and Zn were correlated with the AIx (r = 0.20, *P* = 0.023 and r = 0.20, *P* = 0.026, respectively) in RA patients and with the PWV (r = 0.30, *P* = 0.028) and cIMT (r =  − 0.23, *P* = 0.04) in MetD patients. Furthermore, Fe and Zn were significantly correlated with cIMT (r = 0.26, *P* = 0.039 and r = 0.27, *P* = 0.034, respectively), and Mg was significantly correlated with DIST (r = 0.26, *P* = 0.045) in the control group.

**Fig. 2:**
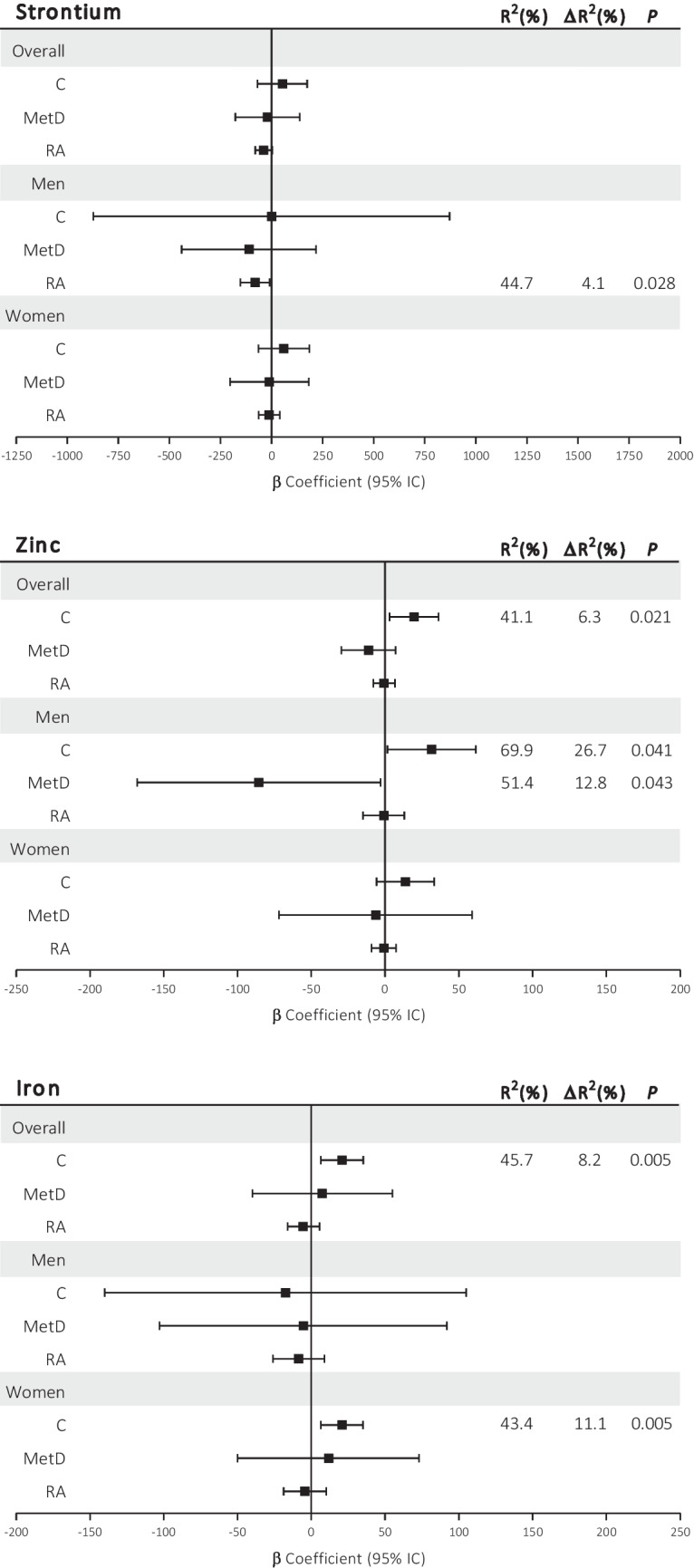
Summaries of the multivariate lineal regression models to estimate the associations between serum levels of strontium (Sr), zinc (Zn), and iron (Fe) and carotid intima-media thickness (cIMT) in the overall cohort and stratified by sex in control participants (C), metabolic disease (MetD) patients, and rheumatoid arthritis (RA) patients. For the final model, the backward method included age, body mass index (BMI), and systolic blood pressure (SBP) for Fe and age, BMI, SBP, and diastolic blood pressure (DBP) for Sr and Zn. Log-transformation was applied for Sr in all groups, and for Zn in the group of men with MetD. *P*-values < 0.05 were considered to indicate statistical significance

To estimate the individual impact of each trace element as an independent predictor of the surrogate markers of CV disease, backward regression models were adjusted (see the “Methods and Patients” section). The varying beta coefficients and their respective confidence intervals (CIs) for the association of each trace element with cIMT are shown in Fig. 2 and Online Resource 3. None of the trace elements showed an association with cIMT in the overall cohort of either RA or MetD patients. However, after stratification of the patients by sex, Sr and Zn were inversely associated with cIMT in men with RA and those with MetD, respectively (Fig. 2). Consequently, increased concentrations of Sr and Zn were associated with decreased cIMT (β =  − 80.95, *P* = 0.028; β =  − 85.70, *P* = 0.043, respectively). Adding Sr to the initial model significantly improved its fit, increasing the explained variance in cIMT by 4.1% (ΔR^2^) to a final R^2^ of 44.7%. Similarly, adding Zn resulted in a significant improvement, with the final model explaining 51.4% (R^2^) of the variance in cIMT, representing a 12.8% (ΔR^2^) increase compared to the initial model. Furthermore, Fe and Zn were found to be independent predictors of cIMT in the control group, implying that increased concentrations of Fe and Zn were associated with increased cIMT (β = 20.83, *P* = 0.005; β = 19.68,* P* = 0.01, respectively). Adding Fe or Zn to the initial models significantly improved the fit by 8.2% and 6.3%, respectively, increasing the final R^2^ to 45.7% and 41.1%, respectively. When the analyses were stratified by sex, the association between Fe and cIMT was confined to women (β = 20.75,* P* = 0.005, ΔR^2^ = 11.1%, R^2^ = 43.4%), while the association between Zn and cIMT was confined to men (β = 31.62,* P* = 0.041, ΔR^2^ = 26.7%, R^2^ = 69.9%) (Fig. 2). In addition, Mg and Se were not associated with cIMT in any of the groups or stratified by sex (Online Resource 1).

**Fig. 3:**
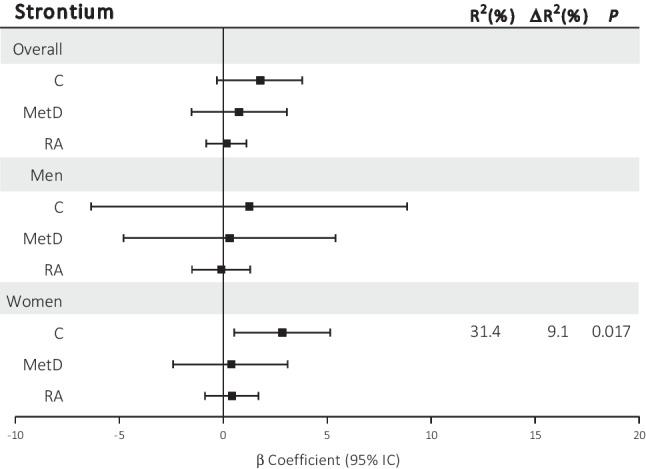
Summaries of the multivariate lineal regression models to estimate the associations between serum levels of strontium (Sr) and pulse wave velocity in the overall cohort and stratified by sex in control participants (C), metabolic disease (MetD) patients, and rheumatoid arthritis (RA) patients. For the final model, the backward method included age. Log-transformation was applied for Sr. *P*-values < 0.05 were considered to indicate statistical significance

Regarding arterial stiffness, there was no association between trace element concentrations and PWV across all groups (Fig. 3 and Online Resource 4). However, when the data were stratified by sex, we found that in control women, higher concentrations of Sr were independently associated with increased PWV values (β = 2.84, *P* = 0.017, ΔR^2^ = 9.1%, R^2^ = 31.4%; Fig. 3).

**Fig. 4:**
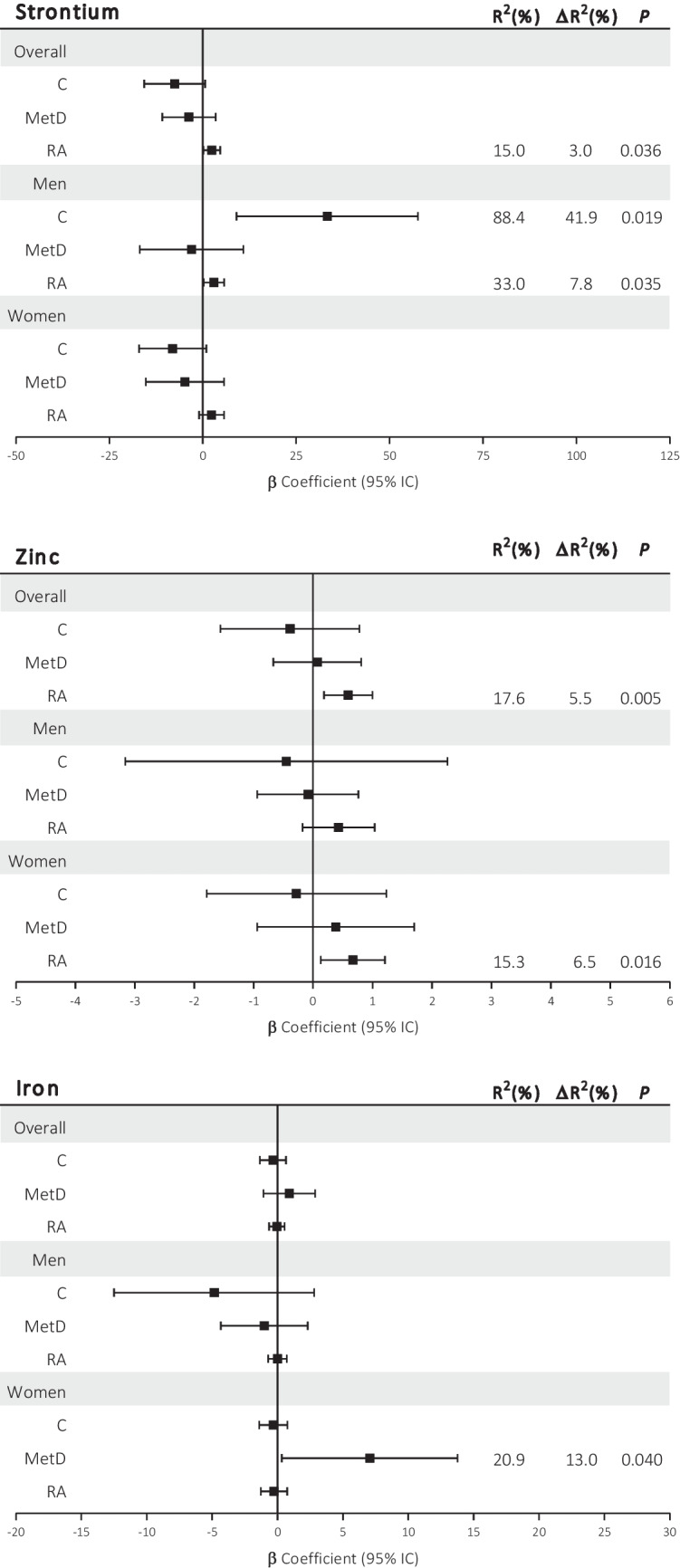
Summaries of the multivariate lineal regression models to estimate the associations between serum levels of strontium (Sr), zinc (Zn), and iron (Fe) and augmentation index in the overall cohort and stratified by sex in control participants (C), metabolic disease (MetD) patients, and rheumatoid arthritis (RA) patients. For the final model, backward method included age, body mass index (BMI), systolic blood pressure (SBP), and diastolic blood pressure (DBP). Log-transformation was applied for Sr in all groups, and for Fe in the group of women with MetD *P*-values < 0.05 were considered to indicate statistical significance

For the AIx (Fig. 4), we showed that Sr and Zn levels were independent predictors of the AIx in patients with RA, showing that increased concentrations of both Sr and Zn were associated with an increased AIx (β = 2.40, *P* = 0.036 and β = 0.059, *P* = 0.005, respectively). Adding Sr or Zn to the initial models significantly improved the fit by 3.0% and 5.5%, respectively, increasing the final R^2^ to 15.0% and 17.6%, respectively. These associations were also observed for Sr in men with RA (β = 2.99, *P* = 0.035, ΔR^2^ = 7.8%, R^2^ = 33.0%) and for Zn in women with RA (β = 0.67, *P* = 0.016, ΔR^2^ = 6.5%, R^2^ = 15.3%). In addition, in control men, Sr levels were independently associated with the AIx, showing that increased concentrations of Sr were associated with an increased AIx (β = 33.33, *P* = 0.019, ΔR^2^ = 41.9%, R^2^ = 88.4%). Furthermore, increased Fe levels were independently associated with an increased AIx in women with MetD (β = 7.05, *P* = 0.040, ΔR^2^ = 13.0%, R^2^ = 20.9%). No other associations were observed for Mg or Se (Online Resource 5).

Finally, as depicted in Online Resource 6 and Online Resource 7, no statistically significant associations were observed between trace elements and DIST or cPP in either the entire study group or when stratified by sex.

## Discussion

In the present study, we investigated the potential associations of selected trace elements with surrogate markers of CVD in the serum of RA patients, aiming to improve the diagnosis and prognosis of CVD in this specific patient population.

Our findings revealed that Sr and Zn concentrations were independently associated with an increased AIx in RA patients. Additionally, Sr levels in male RA patients were independently associated with decreased cIMT. These novel associations between Sr and Zn levels and subclinical atherosclerosis and arterial stiffness markers suggest that these trace elements are potential biomarkers for assessing CVD in RA patients. To the best of our knowledge, this study represents the first report of the associations of Sr and Zn with the AIx and cIMT in RA patients. Considering that cIMT and the AIx are recognized as sensitive surrogate markers of CVD coupled with the observations reported in our study and others of increased values of cIMT and the AIx in RA patients, we believe that our results suggest that measuring the concentration of these trace elements could have clinical utility in identifying RA patients at a greater risk of developing CVD [44–49].

Zn is a relevant element in the context of RA and plays an essential role in both innate and adaptive immune responses, inflammatory regulation responses and bone homeostasis. Similarly, its antioxidant function is important for human health [24, 50]. Nevertheless, conflicting results regarding elevated Zn concentrations in RA patients compared to control participants have been reported [51–54]. Indeed, studies have documented instances of higher, lower, and equal levels, the latter of which aligns with the findings observed in our study. Furthermore, in the general population, an association has been established between lower levels of dietary Zn intake and higher cIMT [55, 56]. However, we did not observe such a relationship in RA patients. Notably, our sex-stratified analysis revealed a significant negative association between Zn and cIMT in male patients within the MetD group. Our results suggest that the impact of Zn on cardiovascular risk may differ between RA and metabolic disorders, emphasizing the importance of considering disease-specific factors when evaluating biomarkers. Furthermore, metabolic disorders are characterized by a lower degree of inflammation, originating from different causes compared to the high-grade autoimmune inflammation seen in RA. Understanding whether Zn’s association with cIMT is influenced by the distinct inflammatory profiles of RA versus MetD could lead to more targeted and effective strategies for managing cardiovascular risk in these populations. On the other hand, Sr has not been involved in processes related to atherosclerosis. However, Sr has been recognized for its use in treating osteoporosis, one of the most common extra-articular complications of RA [57]. Its use is associated with an increased risk of thromboembolic disease, and some studies have shown an association with nonfatal myocardial infarction (MI) [58, 59], although recent studies in osteoporosis patients have not consistently supported these findings. Notably, no studies have evaluated this effect in RA patients. Furthermore, in patients with renal insufficiency, an increase in Sr levels, coupled with elevated calcium levels, may contribute to vascular calcification, a known factor in arterial stiffness [23]. Additionally, our findings revealed higher Sr levels in RA patients with cPP, suggesting a potential role for this element in the atherosclerotic process. Furthermore, we have shown that the Fe concentration is an independent predictor of the AIx in women with MetD. While different studies have reported positive associations between Fe concentrations and cIMT in diverse populations, associations with the AIx have not been described [60–63]. Fe promotes the production of proinflammatory cytokines by T cells, playing a role in the inflammatory response [35]. Surprisingly, our study did not uncover associations between Fe concentrations and surrogate markers of CVD in RA patients. We attribute this result to the high variability observed in serum Fe measurements within our study populations, which may mask the true relationship between Fe levels and cIMT, thereby explaining the absence of these associations. Taken together, these observations underscore the need for further research to elucidate the impact of Zn, Sr, and Fe on cardiovascular health, particularly in the context of RA.

Interestingly, our study also revealed significant differential associations of Fe, Zn, and Sr with cIMT and PWV in control subjects. In particular, we showed that Zn and Fe levels independently predicted cIMT values in these subjects, while Sr and Zn concentrations exhibited positive and negative associations with PWV levels in female control participants. These findings suggest that Zn and Sr may serve as predictors of CVD not only in the presence of inflammation but also in subjects without underlying pathological conditions. However, the mechanism underlying these associations in normal physiology has not been elucidated. Therefore, novel mechanistic and prospective studies are needed to elucidate the involvement of these elements in a nonpathological setting.

Furthermore, despite RA patients presenting lower serum Se concentrations than control participants and men with RA exhibiting significantly lower levels of Mg than MetD patients, our multivariate analyses did not reveal significant associations between Se and Mg concentrations or any of the surrogate markers used in this study. In the literature, decreased serum Se concentrations have been consistently reported in RA patients due to the chronic inflammatory nature of RA, which appears to directly impede Se metabolism [64–67]. Although Se deficiency, owing to its antioxidant role, has been associated with CVD [68], conflicting evidence suggests that elevated Se levels may contribute to an increased incidence of metabolic syndrome, diabetes, and hypertension, with some studies providing inconclusive results [69–71]. However, despite these variations, no significant associations have been found with surrogate markers of CVD in RA patients [33]. Furthermore, despite the documented synergistic anti-inflammatory, antioxidant and immunomodulatory effects of Mg, previous studies have not demonstrated associations between Mg and surrogate CVD markers [72]. Collectively, these findings underscore the intricate relationships of Se and Mg with CV health in the context of RA.

The observed differential associations of these trace elements with PWV, the AIx, and cIMT are of particular interest due to their evaluation of different stages in the atherosclerotic process [73]. Both the PWV and the AIx measure the loss of elasticity in the arteries. While the PWV measures the speed at which the arterial pressure wave travels through the arteries, the AIx measures the reflection of the pressure wave from the peripheral arteries back to the central arteries. As arteries become stiffer, they are less able to expand and contract, imposing greater strain on the heart and increasing the risk of high blood pressure, heart disease, and stroke. Additionally, the PWV directly measures the arterial elasticity of large vessels, while the AIx depends on both peripheral resistance and vascular elasticity [11]. On the other hand, cIMT reflects the thickening of the innermost two layers of the carotid artery wall, providing insight into the early stages of arterial plaque formation before it becomes clinically evident. A surprising result in our study was the lack of association between Sr, Zn, or Fe concentrations and cPP. In contrast to cIMT, cPP results from the thickening of the intima and is considered a late stage in the atherosclerotic process, progressing from the initial fatty streak to an advanced atheromatous plaque [74]. Overall, detecting the presence and extent of subclinical atherosclerosis and arterial stiffness is crucial because it enables early interventions to prevent the progression of the disease and reduce the risk of CV events.

Sex stratification analysis revealed subtle differences between men and women, which could have different explanations. Notably, the prevalence of traditional cardiovascular risk factors differs between the two sexes [75]. Additionally, there is evidence suggesting that RA disease activity is more severe in women than in men, potentially contributing to differential atherosclerotic burdens [76, 77]. In addition, hormonal influences may underlie these sex differences. Estrogens have been shown to decrease the inflammatory immune response, and hormone replacement therapy has been shown to have a beneficial effect on RA disease activity, potentially impacting the progression of atherosclerosis [78].

It is important to emphasize that our study is a cross-sectional observational investigation, limiting our ability to establish causality between Zn, Sr, and Fe levels and CVD risk. Furthermore, another limitation of our study is the imbalance in sample sizes between the experimental groups and the control group. This fact could potentially limit both the generalizability of our findings and our ability to detect significant differences between groups. Overall, our findings suggest potential implications for assessing CVD risk in RA patients; however, prospective and interventional studies with more balanced group sizes are warranted to confirm our observed associations and, more importantly, to determine appropriate levels of Sr, Zn, and Fe for enhancing cardiovascular health in the general population. Such investigations are crucial for our understanding of the intricate interplay between trace elements and CVD risk in RA patients.

In conclusion, our study revealed distinct and significant associations between trace elements (Sr, Zn, and Fe) and various surrogate markers of CVD risk, highlighting their potential role in the clinical management of patients with RA. Understanding these associations would help in assessing CV risk and developing interventions that target both RA-related inflammation and associated CV complications. The differences observed between RA and MetD groups further highlight the complexity of the relationship between trace elements and CV risk, suggesting that these elements may play distinct roles depending on the underlying condition. These findings could have important clinical implications, as monitoring serum levels of trace elements such as Sr, Zn, and Fe might provide valuable insights into the cardiovascular health of RA patients. Incorporating these biomarkers into routine clinical assessments may enhance early detection of CVD risk, allowing for more personalized and timely interventions. Additionally, the methodologies employed in this study, including the evaluation of cIMT and arterial stiffness, offer practical tools that can be integrated into clinical practice to improve CV risk stratification in this high-risk population.

## Supplementary Information

Below is the link to the electronic supplementary material.

Supplementary file1 (DOCX 26 KB)

Supplementary file2 (DOCX 27 KB)

Supplementary file3 (DOCX 35 KB)

Supplementary file4 (DOCX 48 KB)

Supplementary file5 (DOCX 33 KB)

Supplementary file6 (DOCX 56 KB)

Supplementary file7 (DOCX 28 KB)

## Data Availability

No datasets were generated or analysed during the current study.
