# Supplementary material for: Serum Levels of Trace Elements (Magnesium, Iron, Zinc, Selenium, and Strontium) are Differentially Associated with Surrogate Markers of Cardiovascular Disease Risk in Patients with Rheumatoid Arthritis
Source: Biol Trace Elem Res. 2024 Oct 30;203(7):3570–84. doi: 10.1007/s12011-024-04434-8 (PMC12174231; doi:10.1007/s12011-024-04434-8)
Supplement: Supplementary file 1 — Supplementary file1 (DOCX 26 KB) [file 12011_2024_4434_MOESM1_ESM.docx]

**Online Resource 1**. Serum trace element concentration (µg/mL) by groups of non-smokers, smokers and

ex-smokers.

|  | **Non-smoker** | **Smoker** | **Ex-smoker** | ***P*-value** |
| --- | --- | --- | --- | --- |
| **Mg** |  |  |  |  |
| Control | 17.89 (16.18-20.77) | 16.58 (13.96-17.9) | 18.59 (17.13-19.66) | 0.212 |
| MetD | 17.20 (15.14-20.60) | 18.63 (16.24-21.91) | 20.86 (18.60-22.48) | 0.073 |
| RA | 17.43 (16.08-19.22) | 18.06 (15.61-19.03) | 17.38 (15.48-18.72) | 0.721 |
| **Fe** |  |  |  |  |
| Control | 1.14 (0.80-1.40) | 1.69 (0.85-2.35) | 0.97 (0.68-1.45) | 0.200 |
| MetD | 1.07 (0.76-1.36) | 1.49 (1.13-2.01) | 1.71 (1.22-1.96) | a: *P*=0.022 b: *P*=0.009 c: *P*=0.894 |
| RA | 1.02 (0.65-1.48) | 1.08 (0.63-1.45) | 0.97 (0.64-1.47) | 0.991 |
| **Zn** |  |  |  |  |
| Control | 1.53 (0.51-2.69) | 1.80 (1.18-3.94) | 0.30 (0.10-0.90) | a: *P*=0.449 b: *P*=0.012 c: *P*=0.01 |
| MetD | 1.43 (0.57-2.48) | 1.71 (0.45-3.73) | 1.62 (1.24-1.80) | 0.935 |
| RA | 1.12 (0.50-2.73) | 1.12 (0.50-3.34) | 1.34 (0.50-3.09) | 0.898 |
| **Se** |  |  |  |  |
| Control | 0.10 (0.08-0.11) | 0.08 (0.06-0.09) | 0.10 (0.09-0.10) | 0.114 |
| MetD | 0.09 (0.08-0.11) | 0.10 (0.08-0.14) | 0.10 (0.08-0.12) | 0.388 |
| RA | 0.07 (0.06-0.09) | 0.07 (0.06-0.09) | 0.07 (0.06-0.08) | 0.805 |
| **Sr** |  |  |  |  |
| Control | 0.05 (0.03-0.05) | 0.04 (0.03-0.05) | 0.04 (0.03-0.05) | 0.492 |
| MetD | 0.05 (0.04-0.06) | 0.06 (0.04-0.07) | 0.05 (0.03-0.05) | 0.333 |
| RA | 0.04 (0.03-0.06) | 0.03 (0.02-0.06) | 0.04 (0.02-0.05) | 0.522 |

Serum trace element concentration of the control participants (C), metabolic disease (MetD) patients, and rheumatoid arthritis (RA) patients.

*P*-values <0.05 were considered to indicate statistical significance. a: statistical comparison between non-smokers and smokers; b: statistical comparison between non-smokers and ex-smokers; c: statistical comparison between smokers and ex-smokers.
