# Supplementary material for: Serum Levels of Trace Elements (Magnesium, Iron, Zinc, Selenium, and Strontium) are Differentially Associated with Surrogate Markers of Cardiovascular Disease Risk in Patients with Rheumatoid Arthritis
Source: Biol Trace Elem Res. 2024 Oct 30;203(7):3570–84. doi: 10.1007/s12011-024-04434-8 (PMC12174231; doi:10.1007/s12011-024-04434-8)
Supplement: Supplementary file 2 — Supplementary file2 (DOCX 27 KB) [file 12011_2024_4434_MOESM2_ESM.docx]

**Online Resource 2**: Serum trace element concentration (µg/mL) by groups

according to carotid plaque presence.

|  | **Plaque absence** | **Plaque presence** | ***P*-value** |
| --- | --- | --- | --- |
| **Mg** |  |  |  |
| Control | 17.78 (16.21-20.14) | - | - |
| MetD | 18.59 (15.90-21.78) | 16.89 (13.70-20.56) | 0.049 |
| RA | 17.57 (15.79-19.02) | 17.53 (15.78-18.83) | 0.876 |
| **Fe** |  |  |  |
| Control | 1.13 (0.76-1.47) | - | - |
| MetD | 1.17 (0.85-1.44) | 1.15 (0.82-1.64) | 0.971 |
| RA | 1.03 (0.63-1.47) | 0.97 (0.67-1.47) | 0.998 |
| **Zn** |  |  |  |
| Control | 1.51 (0.47-2.69) | - | - |
| MetD | 1.44 (0.51-2.45) | 1.48 (0.67-2.00) | 0.967 |
| RA | 1.06 (0.50-2.74) | 1.32 (0.50-3.21) | 0.341 |
| **Se** |  |  |  |
| Control | 0.09 (0.08-0.11) | - | - |
| MetD | 0.10 (0.08-0.11) | 0.08 (0.07-0.11) | 0.161 |
| RA | 0.07 (0.06-0.09) | 0.07 (0.07-0.09) | 0.480 |
| **Sr** |  |  |  |
| Control | 0.04 (0.03-0.05) | - | - |
| MetD | 0.05 (0.04-0.06) | 0.05 (0.04-0.06) | 0.988 |
| RA | 0.03 (0.02-0.05) | 0.04 (0.03-0.06) | 0.006 |

Serum trace element concentration of the control participants (C), metabolic disease (MetD)

patients, and rheumatoid arthritis (RA) patients. *P*-values <0.05 were considered to indicate statistical

significance.
