# Supplementary material for: Serum Levels of Trace Elements (Magnesium, Iron, Zinc, Selenium, and Strontium) are Differentially Associated with Surrogate Markers of Cardiovascular Disease Risk in Patients with Rheumatoid Arthritis
Source: Biol Trace Elem Res. 2024 Oct 30;203(7):3570–84. doi: 10.1007/s12011-024-04434-8 (PMC12174231; doi:10.1007/s12011-024-04434-8)
Supplement: Supplementary file 4 — Supplementary file4 (DOCX 48 KB) [file 12011_2024_4434_MOESM4_ESM.docx]

**Online Resource 4.** Summaries of the multivariate lineal regression models to estimate the associations between serum levels of magnesium (Mg), iron (Fe), Zinc (Zn), and selenium (Se) and pulse wave velocity in the overall cohort and stratified by sex in control participants (C), metabolic disease (MetD) patients, and rheumatoid arthritis (RA) patients. For the final model, backward method included age, sex, body mass index (BMI), systolic blood pressure (SBP), and diastolic blood pressure (DBP). Log-transformation was applied for Se. *P*-values <0.05 were considered to indicate statistical significance.
