# Supplementary material for: Serum Levels of Trace Elements (Magnesium, Iron, Zinc, Selenium, and Strontium) are Differentially Associated with Surrogate Markers of Cardiovascular Disease Risk in Patients with Rheumatoid Arthritis
Source: Biol Trace Elem Res. 2024 Oct 30;203(7):3570–84. doi: 10.1007/s12011-024-04434-8 (PMC12174231; doi:10.1007/s12011-024-04434-8)
Supplement: Supplementary file 7 — Supplementary file7 (DOCX 28 KB) [file 12011_2024_4434_MOESM7_ESM.docx]

**Online Resource 7.** Summaries of the associations between serum trace element concentration

(µg/mL) and carotid artery plaque presence in the entire cohort and stratified by sex.

|  | **MetD** | | | **RA** | | |
| --- | --- | --- | --- | --- | --- | --- |
|  | **OR** | **CI95%** | ***P*-value** | **OR** | **CI95%** | ***P*-value** |
| **Mg** |  |  |  |  |  |  |
| Overall | 0.886 | 0.763-1.030 | 0.115 | 0.981 | 0.864-1.114 | 0.769 |
| Women | 0.869 | 0.730-1.033 | 0.112 | 0.923 | 0.788-1.081 | 0.320 |
| Men | 0.776 | 0.444-1.356 | 0.373 | 1.138 | 0.898-1.443 | 0.284 |
| **Fe** |  |  |  |  |  |  |
| Overall | 1.109 | 0.415-2.969 | 0.836 | 1.199 | 0.905-1.589 | 0.207 |
| Women | 1.508 | 0.490-4.641 | 0.473 | 1.094 | 0.761-1.572 | 0.761 |
| Men | 0.054 | 0-24.078 | 0.349 | 1.360 | 0.723-2.556 | 0.340 |
| **Zn** |  |  |  |  |  |  |
| Overall | 1.063 | 0.723-1.563 | 0.758 | 1.170 | 0.983-1.392 | 0.077 |
| Women | 1.064 | 0.636-1.780 | 0.813 | 1.105 | 0.900-1.356 | 0.339 |
| Men | 1.045 | 0.450-2.425 | 0.919 | 1.339 | 0.948-1.892 | 0.097 |
| **Se** |  |  |  |  |  |  |
| Overall | 0.052 | 0.001-4.573 | 0.195 | 2.018 | 0.189-21.512 | 0.561 |
| Women | 0.049 | 0-7.044 | 0.235 | 3.872 | 0.205-73.021 | 0.205 |
| Men | 0.043 | 7.416-15.250 | 0.630 | 0.501 | 0.007-37.426 | 0.754 |
| **Sr** |  |  |  |  |  |  |
| Overall | 1.071 | 0.044-26.165 | 0.966 | 1.239 | 0.455-3.378 | 0.675 |
| Women | 1.715 | 0.052-56.418 | 0.762 | 1.317 | 0.369-4.704 | 0.671 |
| Men | 0.014 | 1.128-5.210 | 0.513 | 1.328 | 0.226-7.796 | 0.754 |

Serum trace element concentration in metabolic disease (MetD) patients and rheumatoid arthritis (RA) patients. *P*-values <0.05

are considered to indicate statistical significance. Odds ratios (OR) obtained from regression logistic models. Models were

adjusted for age, sex, body mass index, systolic blood pressure and diastolic blood pressure.
